# Subhepatically located appendicitis due to adhesions: a case report

**DOI:** 10.1186/1752-1947-2-339

**Published:** 2008-10-31

**Authors:** Joseph YS Ting, Rebecca Farley

**Affiliations:** 1 Department of Emergency Medicine, Mater Adult Hospital, Raymond Tce, South Brisbane 4101, Australia; 2 University of Queensland Medical School, Brisbane, Queensland, Australia; 3 Mater Public Hospitals, Raymond Tce, South Brisbane 4101, Australia

## Abstract

**Introduction:**

Acute appendicitis occurs frequently and is a major indication for acute abdominal surgery. Subhepatic appendicitis has rarely been reported and is more difficult to diagnose.

**Case presentation:**

A 71-year-old man with multiple medical comorbidities presented with undifferentiated right abdominal pain. Diagnostic difficulty was encountered due to subhepatic mal-location of the appendix and subsequently atypical presentation for acute appendicitis.

**Conclusion:**

Subhepatic anatomical location of the appendix makes it more difficult to diagnose acute appendicitis at any age, including in older adults.

## Introduction

Acute appendicitis occurs frequently in the community and is usually relatively simple to diagnose in adults [1]. Diagnostic uncertainty due to non-classical evolution of acute appendicitis may occur when the appendix is anatomically mal-located [2]. Subhepatic appendicitis was first described in 1955 by King [3], but has rarely been reported since, and includes a case of delayed diagnosis leading to perforation [4]. Diagnostic difficulty is compounded by old age, with even a normally located acute appendicitis presenting non-classically in the elderly, leading to delayed diagnosis, higher complication and morbidity rates compared with the same condition in young adults [5,6].

## Case presentation

A 71-year-old man was referred by his general practitioner with undifferentiated right abdominal pain which eventually proved to be primary subhepatic appendicitis. The patient presented with sudden severe right upper quadrant pain radiating to the right iliac fossa. He had no nausea, vomiting, anorexia, diarrhoea or fever. The patient had multiple medical comorbidities including moderately severe chronic obstructive pulmonary disease (COPD) and an uncomplicated open cholecystectomy 10 years previously.

The patient's vital observations were BP 96/57 mmHg, pulse 65/minute and regular, temperature 36.5°C, respiratory rate 18/minute and SaO_2 _98% on room air. Abdominal tenderness without rebound or guarding was present only in the right upper quadrant and epigastrium. The abdomen was not distended but obese, there was no palpable mass and normal bowel sounds were present. Occasional basal crepitations were heard on lung auscultation.

**Figure 1:**
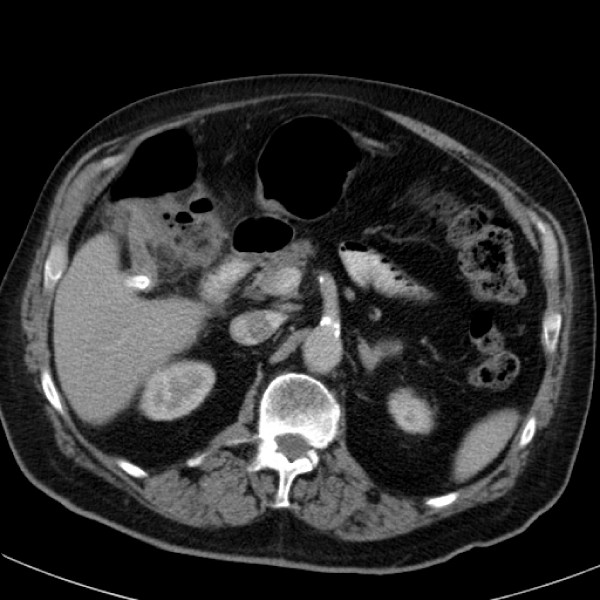
Subhepatic appendicitis with faecolith *in situ*.

Liver function test, lipase and troponin I were normal. His white cell count was elevated at 19.1 (RR 4.5–11 × 10^9^/litre) with predominant neutrophilia 16.66 (RR 1.8–7.7 × 10^9^/litre). There was pre-existing right basal collapse on chest X-ray which was stable in size. Abdominal computed tomography (CT) with oral and intravenous contrast demonstrated a subhepatic appendiceal faecolith. The caecum was rotatedposteriorly andsuperiorlybehind the ascending colon, with the appendix passing superiorly from this to the inferior aspect of the liver wherethe faecolith was located. From there, the appendix passed anteriorlyalongthelowersurfaceof the liverand was associated with inflammation of the hepatic flexure (Figure 1).

The patient underwent laparoscopic appendectomy and adhesiolysis. Operative findings included suppurative subhepatic appendicitis with the ascending colon being adherent to the gallbladder fossa. The histopathology of the resected appendix is that of transmural acute inflammation and focal mucosal ulceration. The patient was ventilated for 72 hours for his COPD and had a 16-day in-patient stay due to surgical wound infection, which was treated with intravenous vancomycin and gentamicin.

## Discussion

This case illustrates the difficulty encountered in trying to diagnose acute appendicitis in patients whose appendix is mal-located due to adhesion or mal-rotation during foetal development [3,4], compounded by the higher risk of non-typical presentation associated with advanced age and multiple comorbidities [5,6]. Subhepatic appendicitis is a rarely reported variant of a common surgical emergency that leads to delayed diagnosis and incurs higher complication rates, including suppuration in this patient and perforation in another patient [4].

In patients aged over 65 years, acute abdominal pain is frequently due to surgical pathology that is difficult to diagnose due to non-typical presentation, including appendicitis [6]. Acute abdominal pain in the elderly therefore presents a challenging diagnostic problem, with lower clinical diagnostic accuracy and poorer outcomes than in younger patients, including for appendicitis [6].

Abdominal CT is frequently required for diagnostic clarification of abdominal pain in the elderly or a non-typical case [5]. The technique is excellent for diagnosing acute appendicitis, with sensitivity being 88–100%, specificity 92–98%, positive predictive value 86–98% and negative predictive value 95–100% [7]. In situations where abdominal CT is inconclusive, clinical diagnosis of appendicitis remains doubtful and the patient remains clinically unwell, a diagnostic laparoscopy is recommended [8].

After cholecystitis, appendicitis is the second most frequent indication for abdominal surgery in late adulthood and the elderly [5]. Multiple comorbidities, complex medication regimens and altered pathophysiological responses contribute to non-typical symptoms and signs as well as delayed diagnosis. Physical findings and investigations in early disease can be misleadingly benign [6]. Although appendicitis is a frequent cause of surgical abdominal disease in late adulthood, it is difficult to diagnose with only half of patients aged more than 50 years receiving the correct diagnosis at first presentation in one prospective study [5].

Diagnostic uncertainties may be compounded by an appendix that is anatomically mal-located [2], as demonstrated in this patient with subhepatic appendicitis. At any age, variation in location of the appendix due to adhesions or developmental anomalies such as foetal intestinal mal-rotation leads to non-typical presentation, delays in diagnosis and increased adverse outcomes [2].

## Conclusion

The relatively high incidence of appendicitis in the general population and the increasing numbers of older adults in the developed world are expected to increase the burden of appendicitis in the elderly. Rarely reported primary subhepatic and other unusually located appendicitis may lead to diagnostic delays at any age, especially in the elderly. Early utilisation of abdominal CT scanning may help in establishing earlier diagnosis.

## Abbreviations

COPD: chronic obstructive pulmonary disease; CT: computed tomography.

## Consent

Written informed consent was obtained from the patient for publication of this case report and any accompanying images. A copy of the written consent is available for review by the Editor-in-Chief of this journal.

## Competing interests

The authors declare that they have no competing interests.

## Authors' contributions

JYST and RF were responsible for the first draft, reviewing subsequent drafts, and approving the final draft(s). Both authors were involved in Emergency Department medical care of the patient and establishing the diagnosis prior to surgical referral.

## References

[B1] Britt H Valenti L Miller G Bayram C Charles J Knox S Henderson J Pan Y Ng A Presentations of abdominal pain in Australian general practice Aust Fam Physician 2004 33 968 969 15630915

[B2] Schumpelick V Dreuw B Ophoff K Appendix and cecum. Embryology, anatomy, and surgical applications Surg Clin North Am 2000 80 1 295 318 10.1016/S0039-6109(05)70407-2 10685154

[B3] King A Subhepatic appendicitis AMA Arch Surg 1955 71 2 265 267 14397970 10.1001/archsurg.1955.01270140113021

[B4] Kulvatunyou N Schein M Perforated subhepatic appendicitis in the laparoscopic era Surg Endosc 2001 15 7 769 10.1007/s004640000027 11592002

[B5] Kraemer M Franke C Ohmann C Yang Q Acute appendicitis in late adulthood: incidence, presentation, and outcome. Results of a prospective multicentre acute abdominal pain study and a review of the literature Langenbecks Arch Surg 2000 385 7 470 481 10.1007/s004230000165 11131250

[B6] de Dombal FT Acute abdominal pain in the elderly J Clin Gastroenterol 1994 19 4 331 335 10.1097/00004836-199412000-00016 7876517

[B7] Leite NP Pereira JM Cunha R Sirlin C CT evaluation of appendicitis and its complications: Imaging techniques and key diagnostic findings AJR 2005 185 406 417 16037513 10.2214/ajr.185.2.01850406

[B8] Ates M Sevil S Bulbul M Routine use of laparoscopy in patients with clinically doubtful diagnosis of appendicitis J Laparoendosc Adv Surg Tech A 2008 18 2 189 193 10.1089/lap.2007.0040 18373441

